# Enhanced transdermal delivery of lidocaine cream via ultrasound and microneedle rollers

**DOI:** 10.3389/fbioe.2025.1612145

**Published:** 2025-07-16

**Authors:** Jiaxiao Sun, Yang Yang, Juanjuan Zheng, Hao Liang, Feng Yan, Wensheng Zhang, Wenqin Xie

**Affiliations:** ^1^ Department of Anesthesiology, Quanzhou First Hospital, Fujian Medical University, Quanzhou, Fujian, China; ^2^ Department of Anesthesiology, West China Hospital, Sichuan University, Chengdu, Sichuan, China; ^3^ Laboratory of Anesthesia and Critical Care Medicine, National-Local Joint Engineering Research Centre of Translational Medicine of Anesthesiology, West China Hospital, Chengdu, Sichuan, China; ^4^ Department of Medical Record, Quanzhou First Hospital, Fujian Medical University, Quanzhou, Fujian, China; ^5^ School of Automation Engineering, University of Electronic Science and Technology, Chengdu, Sichuan, China; ^6^ Department of Ultrasound and Clinical Ultrasound Imaging Drug Research Lab, West China Hospital, Chengdu, Sichuan, China

**Keywords:** lidocaine cream, microneedle roller, transdermal, ultrasound, anesthesiology

## Abstract

**Objective:**

The aim of this study is to determine the most effective physical parameters for optimizing the transdermal delivery of lidocaine cream.

**Methods:**

Preliminary experiments were conducted to optimize ultrasound settings while ensuring safety, guided by visual and histopathological evaluations following observations of skin burns in rats. *Ex vivo* assessments of lidocaine penetration into isolated porcine ear tissue were conducted using Franz diffusion cells and confocal laser scanning microscopy under different intervention conditions *In vivo* analysis involved measuring lidocaine concentrations in rat skin following treatments combining ultrasound and microneedle rollers. The anesthetic efficacy of these interventions was further assessed using the “rat tail-flick test.”

**Results:**

Twelve non-invasive parameter configurations involving ultrasound and microneedle rollers were identified. The combination of ultrasound and microneedle rollers yielded superior results while the ultrasound-only groups demonstrated improved diffusion compared to the control. Notably, ultrasound applied at 260 kHz with a 90% duty cycle, in conjunction with microneedle rollers, achieved the highest diffusion rates, with *ex vivo* cumulative lidocaine permeation at 15 min reaching 45.81 ± 4.19 μg/cm^2^ (vs. baseline 60 min value, p = 0.0017) and microneedle roller alone at 48.62 ± 6.73 μg/cm^2^ (p < 0.0001). Confocal laser scanning microscopy demonstrated minimal lidocaine penetration without interventions, whereas the combined ultrasound and microneedle approach resulted in significantly enhanced penetration, with visible fluorescence deep in the dermis within 5 min. *In vivo* findings corroborated these results, with the combined method facilitating the most rapid onset of anesthesia (mean onset time 28.75 ± 6.41 min, p < 0.05 vs control 67.50 ± 4.63 min) and improved transdermal delivery compared to other groups, achieving 100% anesthetic efficiency rate at 60 min vs. 25% in control.

**Conclusion:**

Microneedle rollers demonstrate superior clinical efficacy over ultrasound, enabling rapid lidocaine delivery (15-min onset *ex vivo*; 32.5-min anesthesia *in vivo*) and achieving 100% anesthetic efficiency for time-sensitive procedures—establishing a practical paradigm shift in transdermal local anesthesia.

## Introduction

Venous cannulation, lumbar puncture, and abscess drainage are commonly performed percutaneous procedures that can cause significant discomfort and pain, particularly among pediatric patients (De Souza et al., 2023; Carvalho et al., 2022). According to recent data, a considerable proportion of adults in the United States, ranging from 11.5 to 66 million individuals, may experience severe needle phobia (Love and Love, 2021). Topical anesthetics, such as the eutectic mixture of local anesthetics cream, are effective in reducing discomfort associated with intravenous and percutaneous procedures. However, the stratum corneum of the skin, characterized by its dense “masonry-like” structure, serves as the primary barrier to transdermal drug delivery (Phatale et al., 2022). For topical anesthetics to achieve full efficacy, they must penetrate to a depth of approximately 3 mm, a process that can take up to 60 min (Kundu and Achar, 2002). This delayed onset renders topical anesthetics impractical in time-sensitive medical environments. Consequently, there is a need to develop safe and effective methods to accelerate the onset of action of these agents.

Ultrasound and microneedle rollers are promising tools for enhancing transdermal drug delivery. These methods facilitate increased medication penetration through various mechanisms and exhibit high potential for interaction. Ultrasound enhances drug permeation by altering tissue structures via thermal and mechanical effects, including pressure, cavitation, and acoustic flow (Aw et al., 2016; Jeong et al., 2021). The impact of ultrasound at frequencies ranging from 20 kHz to 16 MHz has been extensively studied across various drugs (Kost et al., 1989; Mutoh et al., 2003; Boucaud et al., 2001). Since the mid-1990s, research on ultrasound-assisted percutaneous drug delivery has predominantly shifted focus from high to low frequencies (20–100 kHz). Notably, low-frequency ultrasound pretreatment has been reported to significantly accelerate the onset of lidocaine action, reducing the delay from 60 min to as little as 5 min (Watkinson, 2011; Cagnie et al., 2003). However, the details of these findings remain unclear, and no practical clinical applications have been developed thus far. While low-frequency ultrasound (20–100 kHz) enhances skin permeability through cavitation effects, its clinical adoption faces three key limitations. Ultrasound has inconsistent efficacy across skin types and thick or scarred skin requires higher energy doses, increasing thermal injury risk (Polat et al., 2011). In addition, ultrasound-triggered free radicals may degrade protein-based therapeutics (e.g., insulin) during delivery (He et al., 2022).

The application of microneedles prior to ultrasound aims to create a network of microchannels in the stratum corneum, allowing subsequent ultrasound exposure to propel the drug through these channels and enhance its penetration (Han and Das, 2014). Studies have demonstrated the synergistic effects of microneedle pretreatment followed by ultrasound exposure (Petchsangsai et al., 2014; Chen et al., 2010). Conversely, some studies have raised concerns about the potential for air retention within the microchannels created by microneedling, which could hinder drug permeation due to the formation of air pockets in the stratum corneum (Zorec et al., 2015; Park et al., 2010). Microneedle rollers create microchannels to bypass the stratum corneum, yet their utility is constrained. Needle bending during application causes inconsistent penetration depths, leading to erratic drug absorption (Nagra et al., 2022). Residual drug and skin debris in microchannels may trigger infections in immunocompromised patients (Shen et al., 2023). In addition, some studies have reported mild congestion and edema after the application of poly-lactic acid microneedles (Kang et al., 2022).

Alternative transdermal enhancement methods have been widely studied, but each has notable drawbacks. Chemical enhancers like azone and ethanol can irritate the skin and show inconsistent results (Sinha and Kaur, 2000). Iontophoresis requires special equipment and risks burns at high current densities (Abbasi and Heath, 2024). Ablative techniques, such as lasers, are costly and cause thermal damage (Ali-Ahmed et al., 2018). Nanocarriers, including liposomes, improve solubility but have stability issues and low drug capacity (Sudhakar et al., 2021; Opatha et al., 2020). These methods often compromise safety, are expensive, or need complex tools, underscoring the need for practical, non-invasive alternatives for regular clinical use.

Ultrasound and microneedle rollers face significant clinical translation challenges. Ultrasound parameter standardization remains unresolved. Complex interactions between frequency, duty cycle, and power lead to inconsistent outcomes across studies (Polat et al., 2011). This variability hinders device commercialization. Microneedle roller operation suffers from high operator dependency. Variations in rolling pressure and speed cause inconsistent penetration depths, reducing drug delivery reliability (Leone et al., 2018). Synergistic mechanisms are debated that while some report 3.8-fold enhancement with combined approaches (Nayak et al., 2014), others note air pockets from microneedles may block ultrasound energy (Zorec et al., 2015).

The aim of this study was to identify the most effective physical parameters for accelerating the transdermal absorption of topical local anesthetics by comparing their penetration under low-frequency ultrasound and microneedle roller interventions.

## Materials and methods

### Materials

Cylindrical plastic wells (2.2 cm in diameter and 1 cm in height) were custom-made for the experiments. Lidocaine cream (containing 25 mg/g lidocaine and prilocaine) was procured from Ziguang Yipin Pharmaceutical Co., Ltd. (Beijing, China). The Franz diffusion cell and the transdermal drug diffusion tester (model RYJ-6B) were obtained from Huanghai Drug Testing Instrument Co., Ltd. (Shanghai, China). Lidocaine, prilocaine, formic acid, acetonitrile, and calcein were sourced from Sigma-Aldrich (MO, United States). The rat tail light pain tester (model ZH-YLS-12A) was supplied by Zhenghua Biological Instrument and Equipment Co., Ltd. (Anhui, China).

### Animals

Male adult Sprague-Dawley rats, weighing between 225 g and 275 g, were supplied by Dossy Biological Technology Co., Ltd. (Chengdu, China). The animals were housed in groups of five per cage with individual ventilation, unrestricted access to food and water, and controlled temperature and humidity, with lighting conditions set from 7:00 a.m. A total of 386 rats were included in this study. Of these, 258 were used for skin injury experiments, 96 were used for measuring drug concentrations *in vivo*, and 32 were assigned to the tail-flick experiment.

All experimental procedures adhered to the Guide for the Care and Use of Laboratory Animals (NIH Publication No. 80–23, 1996 revision) and strictly followed the biosecurity and institutional safety guidelines established by West China Hospital, Sichuan University (Ethical Approval Number: 20230613003). The cages were arranged on the same rack to minimize the influence of extraneous variables on the study. No animals were excluded from the analysis in this study.

### Porcine ear skins

Porcine ear skin was sourced from Aperture Biotechnology Co., Ltd. Fresh porcine ear skin was rinsed thoroughly and separated from the muscle layer using a surgical blade, ensuring a thickness of no more than 1 mm. The preparation process involved placing the skin on a flat surface, removing the subcutaneous mucosa and adipose tissue with a peeling knife, and adjusting the skin thickness to 0.5–1 mm. The prepared skin was designated for confocal laser scanning microscopy (CLSM) experiments. After processing, the skin was washed twice to ensure cleanliness and maintained in a smooth condition. Each piece of the porcine skin was wrapped in cling film and stored at −20°C.

### Ultrasound and microneedle roller

Low-frequency ultrasound equipment is not commercially available; therefore, this study collaborated with Chengdu University of Electronic Science and Technology to assemble five custom ultrasound systems. These systems operate at fixed frequencies of 1 MHz, 500 kHz, 260 kHz, 40 kHz, and 28 kHz, with the duty cycle set to 10% and input power adjustable between 2 and 200 W.

Microneedle rollers have been used in pharmaceuticals and medical cosmetology for enhancing transdermal drug delivery. For this study, a microneedle roller (manufactured by Guangzhou Kangling Medical Equipment Co., Ltd., China) was selected, featuring needles with a length of 0.2 mm. The roller head was equipped with nine rows of needles, each containing 30 units per row.

## Methods

### Blinding

Animal allocation was conducted in a blinded manner using a random draw conducted prior to the initiation of surgical procedures. To maintain objectivity, separate members of the research team were assigned to handle experimental group assignments, administer interventions, and measure outcome indicators. Group assignments were concealed from the researchers responsible for intervention administration and indicator measurements.

### Experimental parameters of skin damage

During the preliminary experiments, ultrasound parameters with high energy output caused significant damage to rat skin, highlighting the need to identify experimental settings that minimize tissue injury. The screening process was based on the methodology outlined by Draize (1944) (Draize et al., 1944) This phase of the study was divided into two parts: the first involved a preliminary screening to identify ultrasound parameters associated with tissue damage, while the second evaluated the extent of tissue injury resulting from the combination of the selected ultrasound parameters and microneedles, as determined by local histopathological findings.

### Ultrasound parameters of skin damage

The dorsal hair of the rats was removed from a 6 × 6 cm^2^ area using a razor 1 day prior to the experiment. During the procedure, the rats were secured in a flexible fixation frame. The midline of the back was used to demarcate the left and right sides. A circular plastic well was fixed to the left side and denoted with a marker to designate the positive treatment area (L-zone). The corresponding area on the right side served as the negative control (R-zone). The well on the L-zone was filled with lidocaine cream, ensuring there were no gaps. The ultrasound transducer was tightly fitted to the wellhead, and the cream was applied using ultrasound for 5 min, followed by a 1-hour retention period.

Each ultrasound parameter combination was tested on three different rats. If skin damage was observed visually at either 1- or 24-hours post-drug application, the duty cycle was adjusted accordingly. In case of observed damage, the duty cycle was decreased, while in cases of no damage, the duty cycle was increased to a higher energy level. This process continued until the highest combination of ultrasound parameters that caused no visible damage was identified.

The observation index involved a visual assessment of the treated area (L-zone) at 1 and 24 h after drug application, compared to the control area (R-zone). Skin changes, including redness, swelling, discoloration, rash, pus formation, ulceration, or any other adverse reactions, were recorded systematically.

### Ultrasonic and microneedle roller combination of skin damage

The experimental groups are listed in Table 1, with the rat fixation and skin treatment methods consistent with those described in the previous section ultrasound parameters of skin damage. Group 1 involved the application and retention of lidocaine cream for 1 h without additional intervention. Groups 2 through 6 underwent ultrasonic treatment of the L-zone following the procedure detailed in Section ultrasound parameters of skin damage.

**TABLE 1:** Ultrasonic parameters and microneedle roller grouping (*n* = 6).

Group	Processing parameters (Frequency, Duty cycle)	Drugs
1	—	Lidocaine cream
2	Ultrasonic (1 MHz, 40%)	Lidocaine cream
3	Ultrasonic (500 kHz, 40%)	Lidocaine cream
4	Ultrasonic (260 kHz, 90%)	Lidocaine cream
5	Ultrasonic (40 kHz, 100%)	Lidocaine cream
6	Ultrasonic (28 kHz, 100%)	Lidocaine cream
7	Microneedle roller	Lidocaine cream
8	Microneedle roller + ultrasonic (1 MHz, 40%)	Lidocaine cream
9	Microneedle roller + ultrasonic (500 kHz, 40%)	Lidocaine cream
10	Microneedle roller + ultrasonic (260 kHz, 90%)	Lidocaine cream
11	Microneedle roller + ultrasonic (40 kHz, 100%)	Lidocaine cream
12	Microneedle roller + ultrasonic (28 kHz, 100%)	Lidocaine cream

In Group 7, the L-zone was treated with a microneedle roller at a rate of 60 strokes per minute for 5 min, after which a plastic well was fixed, and the same drug treatment protocol was followed. Groups 8 through 12 included a combination of microneedle roller treatment for 5 min in the L-zone, followed by the application of ultrasound for 5 min, with the drug retained for 1 h.

At 1 h, 7 days, and 14 days post-drug exposure, skin samples from the L- and R-zones were collected for pathological examination. The assessment of pathological injury was conducted according to the criteria established by Shackelford et al. (2002).

### Histopathological processing and H&E staining

Collected skin tissues were fixed in 10% neutral buffered formalin (Sigma-Aldrich, United States) for 48 h, dehydrated through graded ethanol series, cleared in xylene, and embedded in paraffin. Sections of 5-μm thickness were cut using a rotary microtome (Leica RM2235). After deparaffinization and rehydration, sections were stained with Harris hematoxylin (Sigma, HHS32; 8 min) and 0.5% eosin Y (Sigma, HT110316; 2 min). Slides were dehydrated, cleared in xylene, and mounted with DPX (Sigma, 06522). Pathological scoring was performed by two blinded investigators using Shackelford’s criteria.

### Franz diffusion cells

The Franz diffusion cell was used to assess drug permeability through the skin. The experimental groups are presented in Table 1. Prior to the experiment, skin samples were thawed for 30 min, rinsed with PBS solution, and their thickness measured with a micrometer to ensure consistency between 0.5 and 1 mm. The skin was positioned between the donor and recipient compartments. The recipient compartment was pre-filled with 7 mL of receptor fluid (PBS solution) and placed on a magnetic stirrer. The compartments were securely fastened to prevent leakage.

Air bubbles between the skin and receptor fluid were removed using a pipette, ensuring no air remained on the skin or liquid surface. The volume of receptor fluid was recorded concurrently. The Franz diffusion cell was then placed in a transdermal drug diffusion tester set to 37°C with constant-temperature water circulation and a rotational speed of 300 rpm.

In Group 1, approximately 5 g of lidocaine cream was added to the donor compartment without leaving space, and the cream was retained for 1 h. Groups 2 through 6 received the same cream application, followed by ultrasound exposure. The ultrasound transducer was positioned 1 cm above the donor compartment and applied for 5 min. For Group 7, the skin was treated with a microneedle roller for 5 min (60 strokes/min) after complete thawing. Groups 8 through 12 underwent microneedle roller treatment for 5 min, followed by 5 min of ultrasound exposure.

**FIGURE 1:**
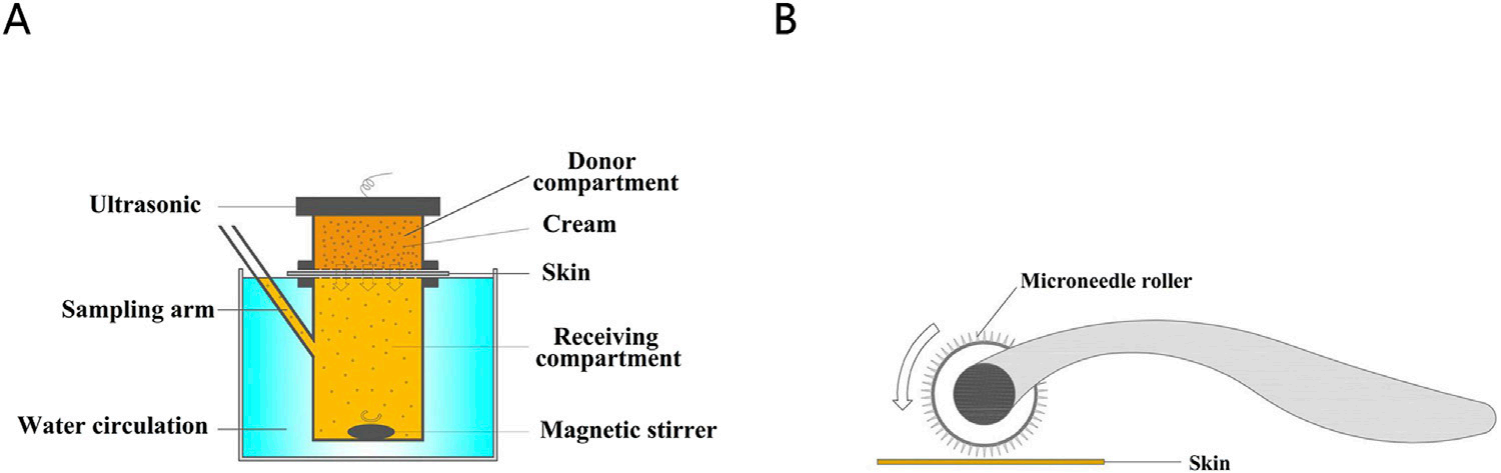
**(A)** The diagram of Franz diffusion pool. **(B)** Treatment of porcine skin with microneedle rollers.

The experimental setup is depicted in Figure 1. Briefly, 200 μL samples were collected at regular intervals from the sampling arm and stored at −20°C. After each sampling, an equivalent volume of receptor fluid was added to the recipient compartment to maintain constant conditions. Concentration data were obtained using liquid chromatography–mass spectrometry (LC-MS/MS). Mean values were calculated from six skin samples.

After determining the concentrations of lidocaine and prilocaine in the receiving compartment using LC-MS/MS, the cumulative drug penetration per unit area for each group was calculated using Equation 1:

$$\left.Q=\left(V\;*\;Cn+\sum_{i=1}^{n-1}Ci\;*\;Vi\right)/A\right)$$ (1)

Specifically, V represents the volume of the receiving compartment; Cn denotes the drug concentration in the receiving compartment; Ci refers to the concentration of the drug in the sample solution; Vi represents the sample size per collection (0.2 mL); and A indicates the skin penetration area (receiving pool opening area, 2.14 cm^2^).

### CLSM

Based on prior experiments, the most significant ultrasound drug penetration effect was observed at a frequency of 260 kHz with a 90% duty cycle. The addition of a microneedle roller further enhanced drug penetration. However, these experiments did not assess the depth of drug penetration. To address this, CLSM was used to qualitatively assess the penetration effects of different interventions by identifying the spatial distribution of the cream within the skin.

The experiment used full-thickness porcine ear skin as the model. Fresh porcine ear skin was rinsed and sectioned without fixation or embedding. Vertical sections (0.5 mm thick) were cut perpendicular to the skin surface using surgical blades at 4°C, then immediately mounted for imaging to minimize post-processing artifacts, divided into four experimental groups:

Group A (Control)

Group B (Microneedle Roller)

Group C (Ultrasound)

Group D (Microneedle Roller + Ultrasound)

Prior to the experiment, Calcein (0.05%) was mixed with lidocaine cream and stored in a light-protected environment for later use. Before the experiment, skin samples were thawed for 30 min and trimmed into 3 × 3 cm^2^ sections. The stratum corneum was positioned upward, and a circular plastic wellhead (1 cm height, 2.2 cm diameter) was adhered tightly to the skin to restrict the movement of the cream.

Group A: The pre-prepared cream (approximately 5 g) was applied within the well and covered with tin foil to protect it from light.

Group B: The skin was pretreated with a microneedle roller at a rate of 60 strokes per minute for 5 min before the cream was applied, and the well was fixed.

Group C: The cream was applied within the well, and an ultrasonic transducer was securely attached to the wellhead to ensure no gap with the cream. Ultrasound was applied for 5 min, after which the area was covered with tin foil.

Group D: The skin was first treated with a microneedle roller for 5 min, followed by the application of the cream and ultrasound treatment for 5 min. The area was then covered with tin foil.

Sampling was performed at 5, 15, 30, and 60 min after the drug came into contact with the skin. During sampling, the skin was rinsed three times with alcohol and physiological saline, and sections were cut perpendicular to the surface using a blade. The samples were placed on glass slides, and the cross-sectional areas were observed under CLSM. Each experiment was repeated thrice.

Skin sections were analyzed using a CLSM (Nikon A1R MP+, Japan) under the following settings: 4 × objective lens, Z-axis imaging, pinhole 1 airy, PMT 700 V, and excitation beam splitter FWTD 488/543/633. All experiments were conducted in a dark environment to prevent interference from ambient light.

The fluorescence images obtained from CLSM were processed and analyzed using NIS-Elements AR Ver5.11.03 software. The background fluorescence of untreated skin was used as a threshold.

### Measurement of drug concentration in skin (*in vivo*)

The metabolism of cream in the skin is a dynamic process influenced by its penetration through the skin and subsequent metabolism by blood circulation. This experiment aimed to assess changes in drug concentration in living animal skin under different parameters.

The experimental groupings and intervention measures were consistent with those outlined in the CLSM section, and the treatment and labeling of rat skin followed the procedures described in section about Ultrasound parameters of skin damage. Rats were anesthetized with isoflurane prior to the experiment. Skin samples were collected at four time points: 5 min (T_1_), 15 min (T_2_), 30 min (T_3_), and 60 min (T_4_) after the application of the cream.

The skin was thoroughly rinsed three times with alcohol pads and saline to remove residual medication from the surface and to prepare the samples. Equal-sized full-thickness skin samples (2 cm in diameter) were excised using a circular blade from the L-zone (drug application area) and the R-zone (control area). Six skin samples were used to calculate the mean values.

### Tail-flick test

As previously described, the radiant heat tail-flick technique was used to assess tail-flick antinociception (Váradi et al., 2015; Kozai et al., 2015). This section focuses on evaluating the onset of local anesthesia induced by lidocaine cream in rats subjected to ultrasound and microneedle interventions.

To establish baseline parameters, the intensity was adjusted to a duration of 2–4 s. Prior to experimental treatments, baseline latencies were recorded for all rats. To prevent tail damage, antinociceptive responses were assessed based on increases in baseline latency, with a maximum allowable latency of 7 s. Data were analyzed using the maximal percent effect (MPE) using the formula: MPE = [(observed latency–baseline latency)/(maximal latency–baseline latency)] × 100%. The onset of anesthesia was defined as the mean time required to achieve an MPE of ≥50% in the rats. The anesthetic efficiency rate (ER) was calculated as the percentage of rats in each group with an MPE of ≥50% at 60 min, relative to the total number of rats in that group. An ER time curve was constructed by plotting the ER of each group at successive measurement intervals.

Thirty-two rats that met the baseline criteria were randomly divided into four experimental groups, following the same grouping protocol described in the CLSM section. The treatment area was the third portion of the rat tail. The experimental conditions were as follows: Group A: The treatment area was evenly covered with compound cream (approximately 5 g). Group B: The skin surface was pretreated with a microneedle roller at 60 strokes/min for 5 min, prior to cream application. Group C: Ultrasound (260 kHz, 90%) was applied for 5 min after cream application, with the ultrasound transmitter positioned 1 cm above the tail. Group D: The skin was pretreated with a microneedle roller followed by 5 min of ultrasound (260 kHz, 90%) after cream application. The onset of anesthesia was assessed at 5-minute intervals for the first 20 min and at 10-minute intervals thereafter, starting from the time of cream application until anesthesia was achieved. Each experimental condition was repeated eight times per group.

### Sample preparation and LC-MS/MS analysis

The sample detection method was adapted from Zhang et al. (2017) Diffusion cell samples were fully thawed and centrifuged at 200,000 × g for 10 min at 4°C. The resulting samples were diluted with ultrapure water in a 1:1 ratio (v/v) before analysis. Skin samples were processed by homogenization using a tissue grinder (KZ-II; Ningbo, China). The tissue homogenate was vortexed with ultrapure water and centrifuged at 10,000 × g for 10 min at 4°C. The supernatant was collected for subsequent analysis. The analysis was conducted using an LC-MS/MS system comprising of an Agilent 6,460 triple quadrupole mass spectrometer with an electrospray ionization source (Agilent Technologies, CA, United States).

Chromatographic separation was achieved using an Agilent Extend C18 column (100 mm × 3 mm, 3.5 μm) at 30°C. An isocratic elution method was used with a mobile phase composed of 0.05% formic acid and acetonitrile at a volume ratio of 84:16, with a flow rate of 0.3 mL/min.

Mass spectrometry conditions included both positive and negative ionization modes with the following settings: sheath gas flow rate, 11.0 L/min, sheath gas heater temperature, 300°C; nebulizer pressure, 45 psi; and capillary voltage, 3,500 V. Lidocaine: m/z 235.1 to 86.1; fragmentation voltage, 115 V; collision energy, 16 V; proparacaine, m/z 221.2 to 86.2, fragmentation voltage, 86 V; collision energy, 8 V. lidocaine-d10 (internal standard): m/z 245.2 to 96.2, fragmentation voltage: 115 V, collision energy: 16 V.

Data acquisition and analysis were conducted using Mass Hunter software (version B.04.00 Build 4.0.479.0, Agilent Technologies, United States).

### Statistical methods

The numerical data are presented as mean ± standard deviation (x ± s). Statistical analyses were conducted using R software (version 4.2.2). Data visualization and figure creation were conducted using GraphPad Prism 8.0, Origin 2022B, CAD 2018, and BioRender Online (https://www.biorender.com).

Single-factor analysis of variance (ANOVA) was applied for comparisons of measurement data across multiple groups. The SNK-q test was used for pairwise comparisons between groups. Variance analysis for repeated measures was used for repeated measurement data. Fisher’s exact probability method was used to compare categorical data between groups.

## Results

### Experimental parameters of skin damage

Table 2 provides the results of naked-eye observations used to assess the ultrasound parameters associated with skin damage.

**TABLE 2:** Ultrasonic parameters with no damage to rat skin (*n* = 3).

Frequency	Duty cycle (%)
1 MHz	40
500 kHz	40
260 kHz	90
40 kHz	100
28 kHz	100

**FIGURE 2:**
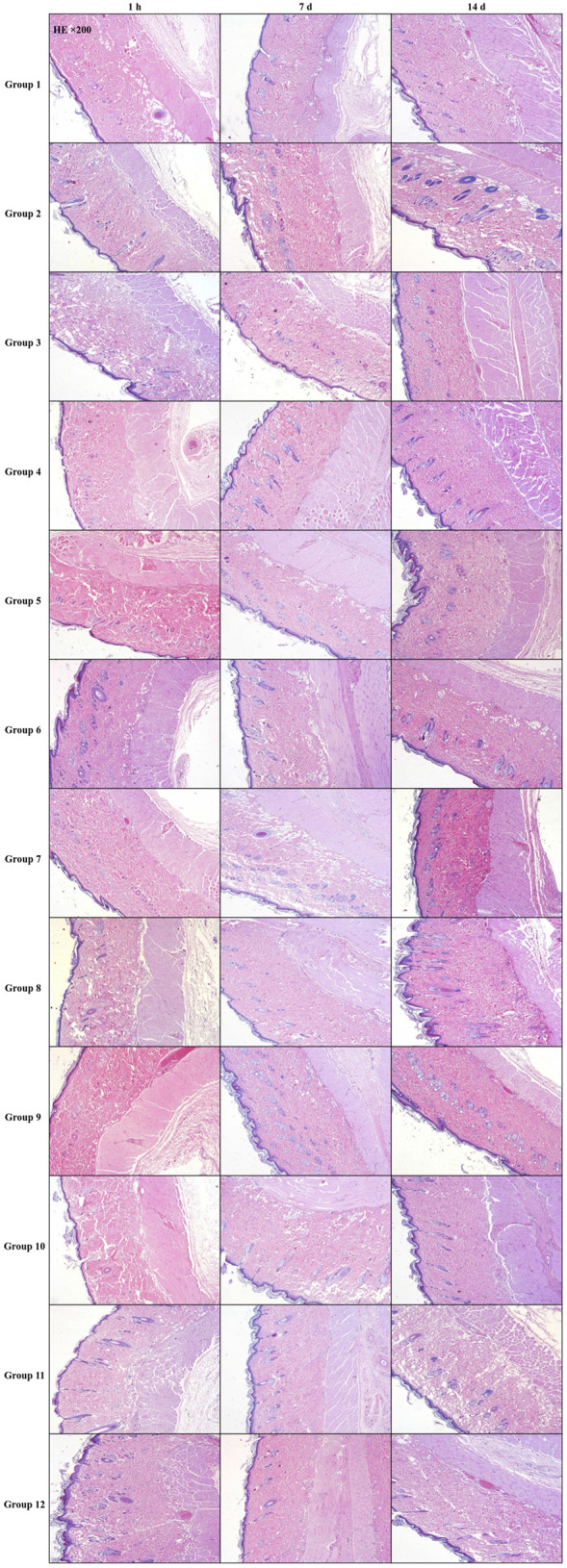
Pathological findings of rat skin stained with HE. n = 6. Scale bar, 200 μm.

To further assess the extent of injury caused by the combination of ultrasound parameters and microneedles, pathological damage scores and detailed findings for each group are summarized in Table 3 and depicted in Figure 2. These findings were derived from local pathological evaluations of the treated skin.

**TABLE 3:** Pathological score of HE staining in rat skin (*n* = 6).

Group	Sampling time point		
	1 h	7 d	14 d
1	0	0	0
2	0	0	0
3	0	0	0
4	0	0	0
5	0	0	0
6	0	0	0
7	0	0	0
8	0	0	0
9	0	0	0
10	0	0	0
11	0	0	0
12	0	0	0

Diagnostic level: 0, normal; 1, slight injury; 2, mild injury; 3, moderate injury; 4, severe injury.

### Franz diffusion cells

**FIGURE 3:**
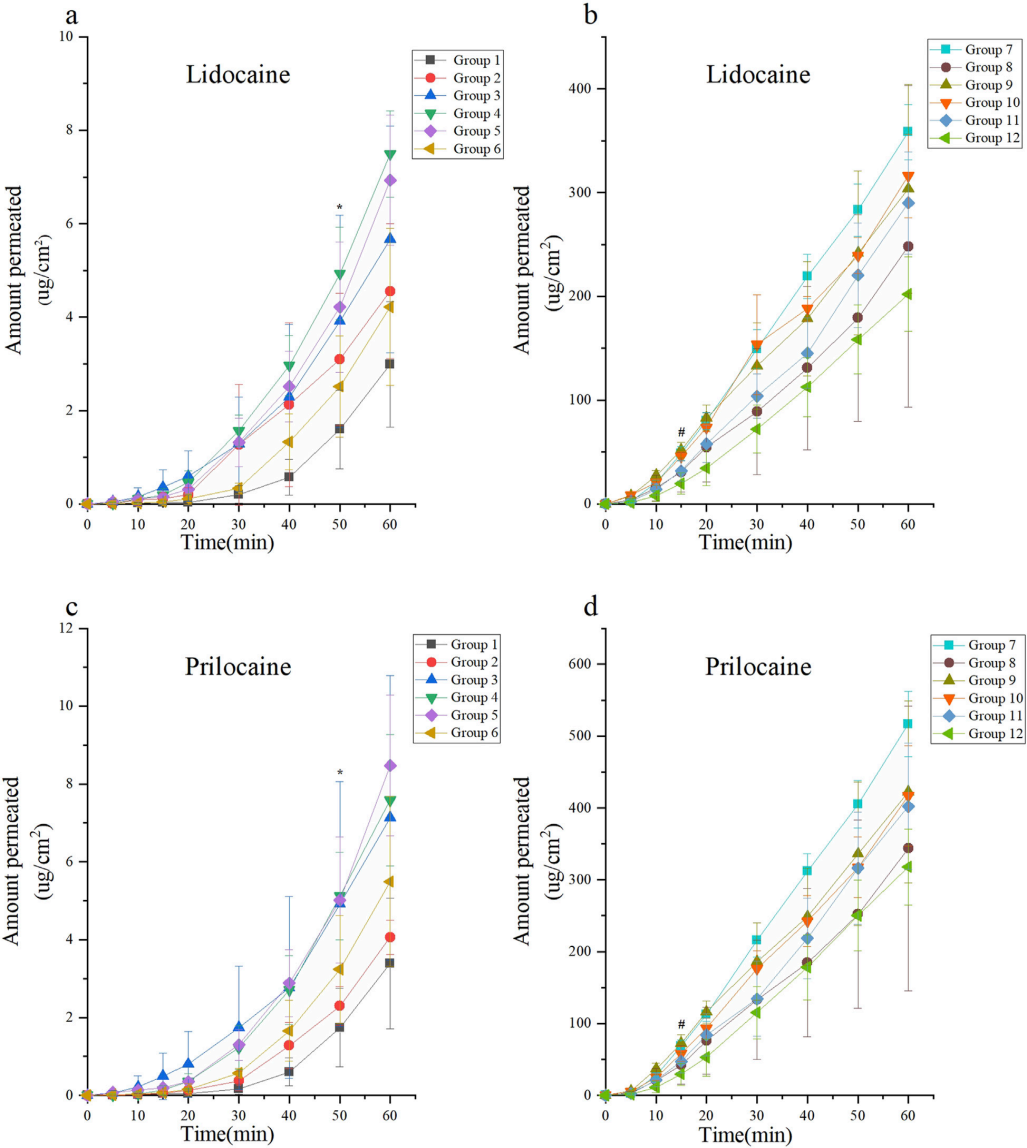
Skin permeability test using the Franz diffusion cell model of lidocaine cream. The skin permeability of lidocaine cream was expressed as μg of lidocaine and prilocaine/cm2 of tissue area. The unit area penetration of lidocaine and prilocaine in each group was compared with the baseline (the unit area penetration of Group 1 at 60 min was considered as the baseline). Lidocaine and prilocaine in group 4 and5 shows a significant difference compared to baseline (*p < 0.05). Lidocaine and prilocaine in groups 7 and 10 showed a significant difference compared to baseline (#p < 0.05). Group 1 was the control group, groups 2 to 6 were the ultrasonic group, group 7 was the microneedle roller group, and groups 8 to 12 were the microneedle roller + ultrasonic group. **(a)** Permeation of lidocaine at each time point over 60 min for Groups 1 to 6. **(b)** Permeation of lidocaine at each time point over 60 min for Groups 7 to 12. **(c)** Permeation of prilocaine at each time point over 60 min for Groups 1 to 6. **(d)** Permeation of prilocaine at each time point over 60 min for Groups 7 to 12.

This experiment evaluated the passive diffusion of lidocaine cream under various intervention conditions. According to Formula 1, the unit area penetration of lidocaine in each group was compared against the baseline, defined as the unit area penetration of Group 1 at 60 min. Lidocaine concentrations were measured and are presented in Figures 3a,b.

Among the groups treated with ultrasound alone (Groups 2–6), the highest diffusion rates were observed in Group 4 (ultrasound: 260 kHz, 90%) and Group 5 (ultrasound: 40 kHz, 100%). In these groups, the diffusion rate at 50 min exceeded the baseline, with diffusion values of (4.94 ± 0.99, *p* < 0.0001; 4.21 ± 1.39, *p* = 0.0166), respectively.

For the microneedle roller intervention groups (Groups 7–12), Group 7 (microneedle roller alone) and Group 10 (microneedle roller + ultrasound: 260 kHz, 90%) demonstrated the fastest diffusion rates. In these groups, the diffusion volume at 15 min was significantly higher than the baseline, with values of (48.62 ± 6.73, *p* < 0.0001; 45.81 ± 4.19, *p* = 0.0017), respectively. The lidocaine cream, a eutectic mixture of lidocaine and prilocaine, exhibited similar diffusion trends for prilocaine as observed for lidocaine. The prilocaine diffusion results are depicted in Figures 3c,d.

### CLSM

**FIGURE 4:**
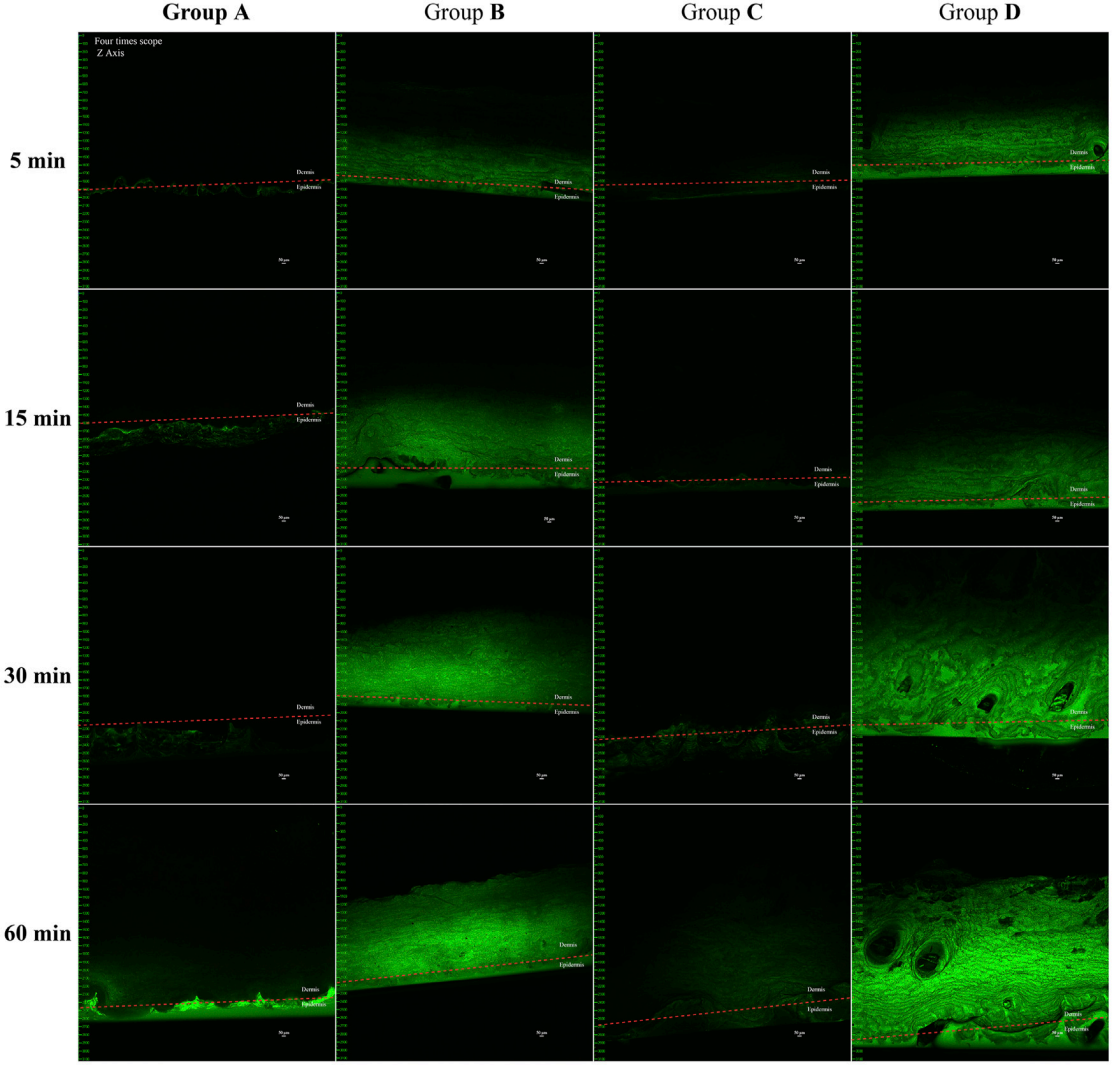
*In vivo* pig ear skin permeation of **(A)** free calcein cream as a negative control, **(B)** microneedle roller, **(C)** ultrasound (260 kHz, 90%), and **(D)** microneedle roller + ultrasonic (260 kHz, 90%). Full-thickness vertical skin sections were prepared with a pathology blade and observed under a confocal microscope for skin-associated fluorescence. The permeation studies lasted for 1 h, and all CLSM images were obtained using a 4× objective. Up, dermis; down, epidermis. Scale bar: 50 μm.

The penetration depth of lidocaine cream in the four experimental groups—(A) control, (B) microneedle roller only, (C) ultrasound only, and (D) microneedle roller + ultrasound—is depicted in the cross-sectional images presented in Figure 4.

Group A: Fluorescence aggregation was observed only on the skin surface at 5, 15, and 30 min. Weak fluorescence signals in the dermis were not discovered until 60 min, indicating that without assistance, the cream minimally penetrated beyond the epidermis.

Group C (Ultrasound: 260 kHz, 90%): Similar fluorescence patterns to Group A were observed at 5 and 15 min. By 30 min, faint fluorescence appeared below the stratum corneum, and at 60 min, noticeable fluorescence signals were present in the dermis, demonstrating that ultrasound had a measurable permeation-enhancing effect.

Groups B and D: Both groups exhibited significant fluorescence in the dermis within 5 min, indicating rapid drug penetration facilitated by the microneedle roller. The differences in fluorescence intensity between the two groups at this time point were minimal.

Comparison of fluorescence intensity at 60 min: group D ≥ group B > group C > group A.

### Drug concentration *in vivo*

**FIGURE 5:**
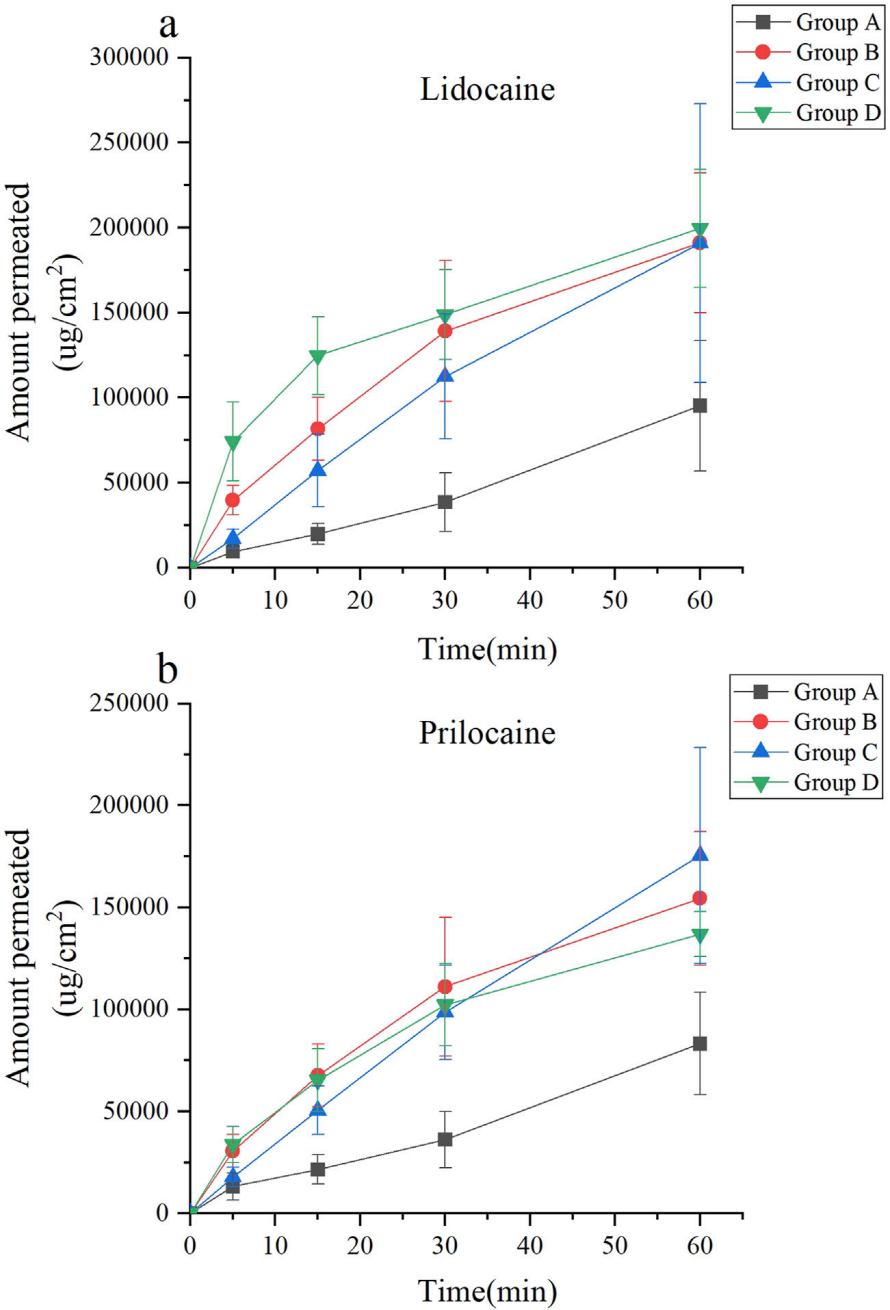
Drug concentration of lidocaine cream *in vivo* under different combinations of parameters. **(a)** The concentration of lidocaine. **(b)** The concentration of prilocaine.

LCMS/MS analysis was used to quantify local anesthetics in the skin and assess the *in vivo* penetration of lidocaine cream. Figure 5 depicts the results of comparing the transdermal penetration of lidocaine cream between the experimental groups at different time points and the baseline (group A’s skin penetration at 60 min). The concentration of lidocaine was measured in groups B, C, and D. Results revealed that at 30 min, the skin drug concentration penetration was higher than the baseline in groups B (*F* = 18.57, *p* < 0.0001) and D (*F* = 16.04, *p* < 0.0001). At 60 min, skin drug concentration penetration was higher than that at baseline in group C (*F* = 12.72, p < 0.0001). When measuring the local anesthetic substance as procaine, the skin drug concentration penetration of groups B, C, and D at 60 min was higher than baseline (*F*_*B*_ = 20.58, *p*_*B*_ < 0.0001; *F*_*C*_ = 25.60, *p*_*C*_ < 0.0001; *F*_*D*_ = 30.49, *p*_*D*_ < 0.0001).

### Tail-flick test

**FIGURE 6:**
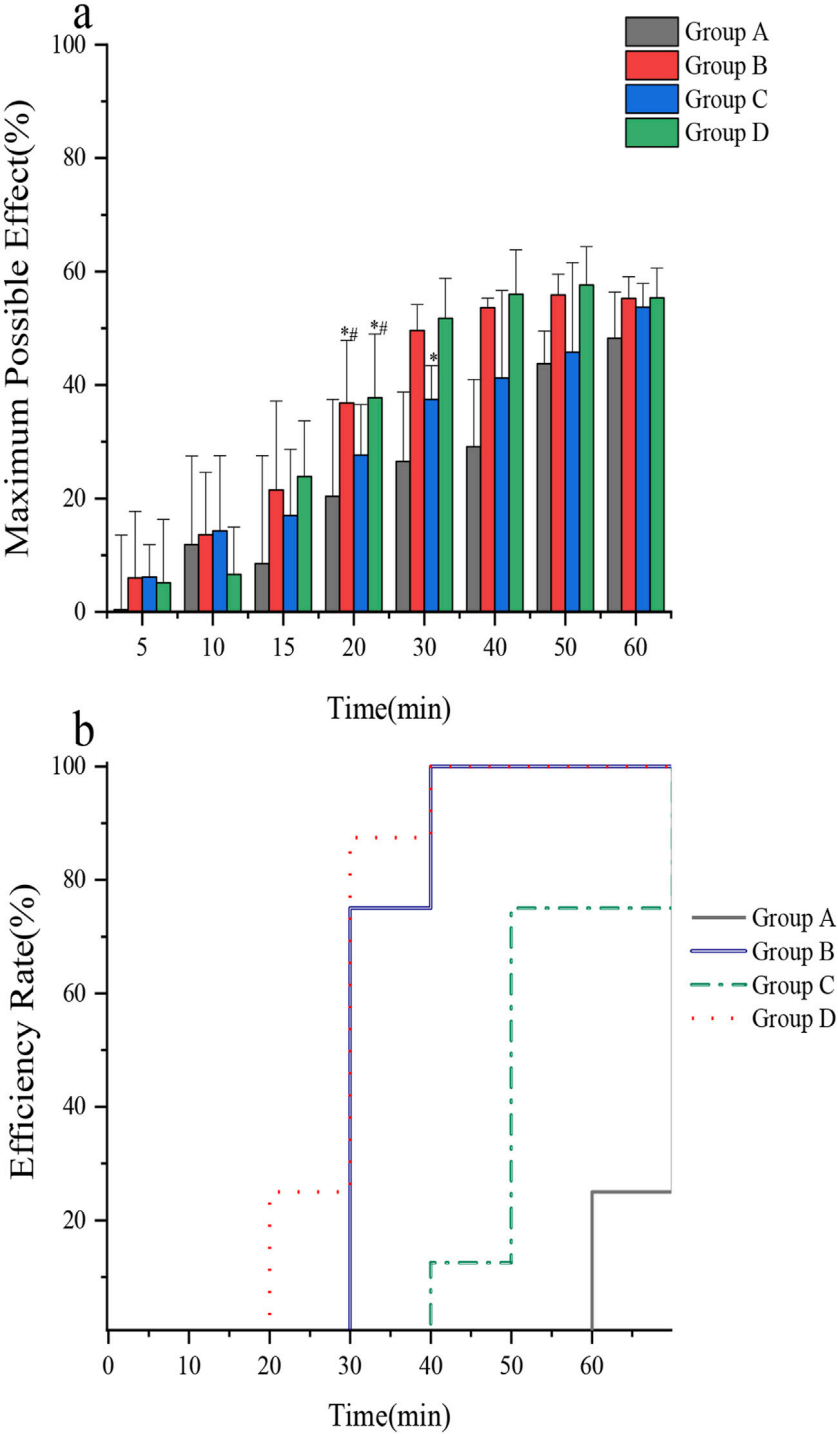
**(a)** Comparison of the maximum possible effect of tail skin in rats; **(b)** Comparison of anesthetic efficiency of tail skin in rats. *In vivo* rat tail skin permeation of (A) control group, (B) microneedle roller, (C) ultrasound(260 kHz, 90%), and (D) microneedle roller + ultrasonic (260 kHz, 90%).

The tail-flick test was used to assess the analgesic efficacy of lidocaine cream using different intervention methods. The Fisher’s exact probability method indicated that the onset rate of group A was lower than that of groups B and D at 60 min (*p* = 0.01). Additionally, a single-factor analysis of variance revealed that the average onset time of rats varied across the four groups (*F* = 47.71, *p* < 0.0001). Further analysis using the *SNK-q* test revealed that the onset time of anesthesia in group A was higher than that in groups B, C, and D. Group C exhibited a longer onset time than groups B and D. Concurrently, no significant difference was found between groups B and D (Table 4). The MPE of rats in groups B and D was higher than that in groups A and C 20 min after application (*F* = 3.52, *p* = 0.0278). Additionally, the MPE of rats in group C was significantly higher than that in group A 30 min after application (*F* = 17.21, *p* < 0.0001). However, there was no significant difference in the MPE of rats in groups A, B, C, and D 60 min after application (*F* = 2.87, *p* = 0.0543) (Figure 6a). The ER of the tail skin of rats from each group is depicted in Figure 6b.

**TABLE 4:** The effect of tail skin anesthesia in rats (*n* = 8).

Group	Effective cases	60 min ER (%)	Average time (min)a
A	2	25	67.50 ± 4.63
B	8	100^*^	32.50 ± 4.63^*$^
C	6	75	52.50 ± 11.65^*^
D	8	100^*^	28.75 ± 6.41^*$^
F	-	-	47.71
P	-	0.001	<0.0001

Note: ER, efficiency rate; a, The average time from the beginning to the complete onset of anesthesia in all rats in each group; *: Compared with group A, P < 0.05; $: Compared with group C, P < 0.05.

## Discussion

The frequency of ultrasound plays a significant role in determining its biological effects on skin tissue, with thermal effects intensifying as both frequency and exposure duration increase (Ahmadi et al., 2012). The potential for adverse outcomes, such as burns, exfoliation, and tissue necrosis, necessitates careful consideration. Managing heat accumulation during ultrasound application is essential for mitigating risks. Research has demonstrated that high-output ultrasonic energy can induce skin injuries in rats, highlighting the critical need to establish safe ultrasound parameters for skin-related studies (Miller et al., 2012). A two-phase study was undertaken to identify ultrasound parameters that do not cause damage to the skin. In the first phase, visual inspections were conducted to screen for skin damage, and in the second phase, these parameters were combined with microneedle interventions. Histopathological analysis ultimately identified 12 parameter combinations that did not cause damage, which can be used in future experiments.

Compound lidocaine cream, a topical analgesic, contains a eutectic mixture of 2.5% lidocaine and prilocaine. This formulation lowers the melting point of the mixture, enhancing thermodynamic activity and increasing skin permeability (Marwah et al., 2014). As revealed by the manufacturer, the onset time of the drug is approximately 60 min. Thus, the permeation rate per unit area of the diffusion cell at 60 min was used as the baseline in this study.

The findings demonstrated that within the ultrasound-only groups (Groups 1–6), the transmission of lidocaine per unit area was significantly higher in Groups 4 (ultrasound: 260 kHz, 90%) and 5 (ultrasound: 40 kHz, 100%) at 50 min compared to the baseline (*p* < 0.0001; *p* = 0.0166). By 60 min, the remaining ultrasound-only groups exhibited higher diffusion rates than the baseline. Groups that combined ultrasound with microneedles (Groups 7–12) achieved greater diffusion compared to the ultrasound-only groups. Notably, Group 7 (microneedle only) and Group 10 (microneedle + ultrasound: 260 kHz, 90%) revealed significantly higher diffusion rates than the baseline as early as 15 min (*p* < 0.0001; *p* = 0.0017). The permeability results for prilocaine were consistent with those observed for lidocaine, indicating similar *ex vivo* transdermal properties for these two local anesthetics.

Ultrasound enhances transdermal delivery through thermal effects (Azagury et al., 2014) (increasing lipid fluidity and widening cell junctions) (Machet and Boucaud, 2002)and mechanical effects (Kaushik and Keck, 2021; Tezel et al., 2002), particularly cavitation (generating microchannels via bubble collapse)) (Tang et al., 2002). While these mechanisms theoretically favor lower frequencies and higher power for stronger cavitation (Frenkel, 2008; Boucaud et al., 2002), The strength of the cavitation effect is largely influenced by ultrasound frequency and power, with lower frequencies and higher power generally producing stronger effects, theoretically enhancing transdermal drug delivery. (Pereira et al., 2017; Liu et al., 2006; Kim et al., 2007). Our Franz cell results unexpectedly showed optimal efficacy at 260 kHz and 40 kHz within the ultrasound-only groups, reducing the onset time by only 10 min compared to control.

However, findings from the Franz diffusion cell experiments in this study deviate from this theoretical understanding. In the ultrasound-only groups, parameters of 260 kHz and 40 kHz yielded the best performance, reducing the time to reach baseline penetration by only 10 min. Ultrasound alone did not significantly enhance the skin permeation of lidocaine cream. Additionally, the thermal and cavitation effects observed in these groups were not the strongest among the five tested ultrasound parameter combinations. The complexity of the concurrent effects of the ultrasound makes it challenging to isolate and measure the contribution of each factor. This lack of clarity in the underlying ultrasound mechanisms of has hindered advancements in ultrasound-mediated transdermal drug delivery (Seah and Teo, 2018).

In recent years, ultrasound and microneedles have emerged as effective physical enhancement methods for skin penetration. Ultrasound enhances skin permeability through thermal, mechanical, and cavitation effects, while microneedles create pores on the skin surface, facilitating drug passage. Research indicates that the concurrent application of ultrasound and microneedles results in synergistic effects, notably enhancing the transdermal penetration of drugs. For instance, Chen et al. (2010) demonstrated that the combined use of ultrasound and microneedles notably boosts the transdermal penetration of macromolecular drugs like bovine serum albumin. Similarly, Nayak et al. (2014) observed a 3.8-fold increase in the transdermal penetration of the local anesthetic lidocaine when microneedles were paired with low-frequency ultrasound. These studies underscore the significant enhancement in skin penetration achieved through the combined application of ultrasound and microneedles, particularly in delivering macromolecular drugs. However, the findings of this study deviate from existing literature in certain aspects. While the combined application of ultrasound and microneedles exhibited enhanced transdermal penetration compared to Nayak et al., our study revealed that microneedles alone demonstrated significant superiority, with the incremental impact of ultrasound being relatively modest. This discrepancy could be attributed to the specific ultrasound parameters utilized in our study (such as frequency and power) potentially failing to fully exploit the synergistic potential of ultrasound. Moreover, the efficacy of microneedle penetration might have already surpassed the threshold required to breach the skin barrier, limiting the scope for additional enhancement through ultrasound. These variations may stem from disparities in experimental methodologies and standardization issues concerning ultrasound equipment, underscoring the need for future investigations to optimize ultrasound parameters and delineate the optimal conditions for leveraging the combined approach of ultrasound and microneedles.

Moreover, previous studies have rarely detailed the experimental setups and the specific impact of ultrasound parameters, likely due to the multitude of variables influencing transdermal ultrasound outcomes. The efficacy of ultrasound for transdermal drug delivery is influenced by numerous factors, including the mode of energy dispersion, size and type of ultrasound transducer, ultrasound parameters (power, frequency, duty cycle), treatment duration, characteristics of the coupling medium (thickness and type), and skin sample properties (treatment conditions, thickness variability, sampling area size, and skin species type). These variables can significantly change the penetration efficacy of ultrasound, contributing to inconsistent findings across studies. Additionally, inherent uncertainties in the ultrasound system itself may further affect diffusion results.

Microneedle rollers effectively bypass the principal barrier, the stratum corneum (Polat et al., 2010; Han and Das, 2013), creating microchannels that enable rapid drug delivery to the epidermis and dermis. This mechanism explains the superior performance of microneedle interventions observed in our Franz cell, CLSM, and *in vivo* experiments, achieving therapeutic penetration within 15 min *ex vivo* and rapid anesthesia onset *in vivo* (32.5 min).

Research by Nayak et al. demonstrated that ultrasound alone, when applied for 2 h, did not significantly enhance the penetration of lidocaine gel into porcine skin (Nayak et al., 2014). However, microneedle application substantially increased lidocaine penetration, achieving therapeutic levels within 9 min. The study reported a 2.8-fold increase in penetration with microneedles compared to passive diffusion and a 3.8-fold increase when microneedles were combined with ultrasound. The diffusion cell analysis revealed that all parameter combinations involving both ultrasound and microneedle rollers (Groups 7–12) exhibited significantly higher skin permeation compared to the ultrasound-only groups. These findings indicate that microneedle rollers are more effective than ultrasound alone in enhancing the permeation of lidocaine cream.

While alternative transdermal enhancement strategies exist (e.g., chemical enhancers, iontophoresis, ablative techniques, nanocarriers) (Sinha and Kaur, 2000), their clinical application is often limited by safety concerns, cost, complexity, or delayed onset, as previously noted. This underscores the ongoing need for practical, non-invasive alternatives suitable for routine clinical settings.

However, in groups where microneedle rollers were used, the addition of ultrasound did not result in a statistically significant increase in permeation compared to groups without ultrasound. This observation diverges from the outcomes of previous studies. Two potential reasons may account for this discrepancy. First, the parameters of the customized ultrasound experimental platform used in this study differed from those used in earlier experiments. Second, the extent of lidocaine cream penetration achieved by microneedle rollers may surpass the level attainable through ultrasound alone, potentially overshadowing the ultrasound effect. Further research is necessary to address this issue.

The Franz diffusion cell is a widely used method for assessing skin pharmacokinetics and offers a robust platform for large-scale screening of potential target factors (Supe and Takudage, 2020). For local anesthetic agents to provide effective analgesia, they must penetrate the skin and accumulate in dermal nerve tissue, where they inhibit sodium ion channels on sensory neuron cell membranes and pathways. To assess the extent of drug penetration under various interventions, the most effective experimental parameter identified in the Franz diffusion cell experiment was used to track the position of calcein fluorescence in different carriers via CLSM (Adin et al., 2024; Wang et al., 2013; Alvarez-Román et al., 2004).

The fluorescence intensity comparison at 1 h, as presented in Figure 4, demonstrated the following hierarchy: Group D exhibited the highest intensity, followed by Groups B, C, and A. These findings indicate that ultrasound enhances fluorescence diffusion through the skin, although microneedling exhibited a more pronounced effect. The results were consistent with the Franz diffusion cell findings in the *ex vivo* permeation study.

Despite these findings, there are several limitations which must be acknowledged: CLSM analysis provides qualitative rather than quantitative insights into skin permeation. CLSM can only observe permeation at specific time points and cannot capture the dynamic process of skin permeation. Calcein, as a fluorescent marker, does not bind to the active ingredients in lidocaine cream and can only indirectly indicate penetration depth.

The simulated physiological transdermal environment in isolated experiments is subject to greater variability and external influences, making it less reliable compared to *in vivo* animal studies. To address the inability of CLSM to perform quantitative analysis, the same parameter interventions were applied to living rats to assess changes in lidocaine cream content within skin samples over different time points.

For a topical local anesthetic to effectively reduce pain, its active ingredient must penetrate and remain within the dermis. However, as the anesthetic reaches the dermis, systemic absorption into the bloodstream becomes inevitable, leading to continuous metabolism of the drug in living skin.

In the *in vivo* results for lidocaine, using the baseline defined as the skin permeation of Group A at 60 min, the following findings were observed:

At 30 min, the permeation in Groups B (microneedle roller) and D (microneedle roller + ultrasound) was significantly higher than the baseline.

At 60 min, the permeation in Group C (ultrasound alone) exceeded the baseline.

These results align with the trends observed in previous experiments. However, when prilocaine was assessed, all groups demonstrated higher permeation than the baseline at 60 min. This extended sampling period should therefore be addressed.

Topical anesthetic creams must penetrate to a depth of approximately 3 mm to achieve full effectiveness on the skin (Taddio et al., 2016). Although numerous animal models are available for studying pain, few are specifically suited for evaluating transdermal analgesia with local anesthetics. The cutaneous trunci muscle reflex, commonly used to assess animal responses to noxious stimuli, was initially considered appropriate for this purpose (Petruska et al., 2014). However, preliminary experiments in this study demonstrated its limited use, as transdermal local anesthetics only partially inhibit nociceptive nerves, making it challenging to differentiate nociceptive responses from tactile sensations in animals. Further validation of alternative models is therefore required.

To address these limitations, this study utilized the tail-flick test in rats to assess the transcutaneous analgesic effects of lidocaine cream under different intervention parameters (Bhowmik et al., 2021). The findings indicated the following trends: The average onset time of anesthesia followed the order: Group B (microneedle roller) = Group D (microneedle roller + ultrasound) < Group C (ultrasound alone) < Group A (control). At 60 min, the anesthetic effectiveness was ranked as: Group B = Group D > Group C > Group A. These results are consistent with previous experimental findings.

This study has several limitations.

1 Ultrasound parameters: Due to technical constraints, the handmade ultrasound devices used in the study could only operate with fixed power settings and variable duty cycles. However, ultrasound power is a key factor influencing cavitation and thermal effects, both of which may significantly affect the transdermal delivery of lidocaine cream. Future research is required to systematically examine the role of power in enhancing transdermal drug delivery. Additionally, experimental ultrasound platforms are complex and costly, which may limit their broader application.
2 Microneedle roller treatment: To ensure consistency, the same researcher controlled the rolling frequency during microneedle treatment. However, the applied pressure could not be precisely quantified or standardized, potentially introducing experimental variability.
3 *Ex vivo* skin model: The *ex vivo* experiments utilized porcine ear skin, but several factors may have influenced the outcomes, including differences in skin extraction sites, the time elapsed since extraction, and variations in skin moisture levels. These variables could change the drug penetration characteristics and limit the generalizability of the findings.
4 Lack of post-intervention follow-up: The study did not include a follow-up assessment of the skin post-intervention, precluding the evaluation of potential long-term effects on skin health and barrier function. Hence, this omission does not address the potential for unintended adverse outcomes.

Further investigations are needed to address these limitations, particularly regarding the optimization of ultrasound parameters, standardization of microneedle roller techniques, refinement of *ex vivo* models, and evaluation of long-term skin effects. These steps will help to improve the reliability and applicability of transdermal drug delivery research.

## Conclusion

This study systematically evaluated physical enhancement strategies to overcome the critical limitation of delayed onset (≥60 min) in topical lidocaine anesthesia. While low-frequency ultrasound (260 kHz, 90% duty cycle) demonstrated a measurable improvement over passive diffusion (reducing *in vivo* onset time to 52.5 ± 8.70 min vs. 67.50 ± 4.63 min in control, p < 0.05), microneedle rollers (0.2 mm needles) proved markedly superior. *Ex vivo*, microneedle rollers alone achieved therapeutic lidocaine permeation within 15 min (48.62 ± 6.73 μg/cm^2^ vs. baseline, p < 0.0001). Critically, *in vivo* testing confirmed microneedle rollers enabled rapid anesthesia onset (32.5 ± 4.63 min) and achieved 100% anesthetic efficiency at 60 min. The addition of ultrasound to microneedle rollers offered no statistically significant benefit over microneedle rollers alone. Therefore, microneedle rollers represent the optimized clinical paradigm for accelerated transdermal lidocaine delivery, providing a rapid, reliable, and practical solution for time-sensitive procedures. Further multi-center clinical study investigating clinical efficacy of microneedle rollers in optimizing transdermal local anesthesia across skin types are needed, the results of which may advocate regular clinical use of microneedle rollers in optimizing transdermal local anesthesia.

## Data Availability

The original contributions presented in the study are included in the article/supplementary material, further inquiries can be directed to the corresponding authors.

## Abbreviations

(CLSM), confocal laser scanning microscopy; (LC-MS/MS), liquid chromatography–mass spectrometry; (MPE), the maximal percent effect; (ER), efficiency rate; (x ± s), the mean ± standard deviation.
